# Late-stage primary renal angiosarcoma: an extremely rare cancer complicated by COVID-19 post-operatively

**DOI:** 10.1093/jscr/rjab562

**Published:** 2021-12-24

**Authors:** Reilly Carr, Hannah Moreland, Michael Hsueh-Ching Hsia

**Affiliations:** University of South Carolina School of Medicine, Columbia, SC, USA; University of South Carolina School of Medicine, Columbia, SC, USA; Department of Urology, Medical University of South Carolina, Florence, SC, USA

## Abstract

Primary renal angiosarcoma (AS) encompasses only 1% of soft tissue sarcomas, and it is seldom seen in literature. Because of its rarity, few risk factors for its development have been established. Due to lack of screening guidelines for kidney cancer, it is often found late when patients become symptomatic. We present the case of a male patient who presented with gross hematuria and flank pain and was discovered to have a large renal mass. Following successful resection, pathology showed it to be AS. The patient had a post-operative course complicated by two separate COVID-19 infections and expired 200 days after surgery. This case not only highlights an extremely rare renal cancer but also illustrates the challenges patients with complex medical issues faced in the era of COVID-19.

## INTRODUCTION

Angiosarcoma (AS) is a rare, endothelial cancer that forms in the linings of blood vessels and lymphatics and comprises <1% of all soft-tissue sarcomas [1]. Given its endovascular origin, AS can develop anywhere, but involvement of the kidney usually indicates metastasis with widespread systemic disease [2]. AS primary to the kidney is exceedingly rare, so very few cases have been reported in the literature, with most occurring in male patients who are in the sixth and seventh decades of life [3]. Risk factors for AS include prior radiation and exposure to arsenic, thorium dioxide and vinyl chloride, but renal-specific risk factors have not been identified [4]. AS presents similarly to renal cell carcinoma (RCC), with the most common symptomatic presentation being flank pain and gross hematuria [5]. AS and RCC cannot be differentiated with imaging, thus diagnosis can only be made with histologic examination and immunohistochemical staining following resection.

**Figure 1:**
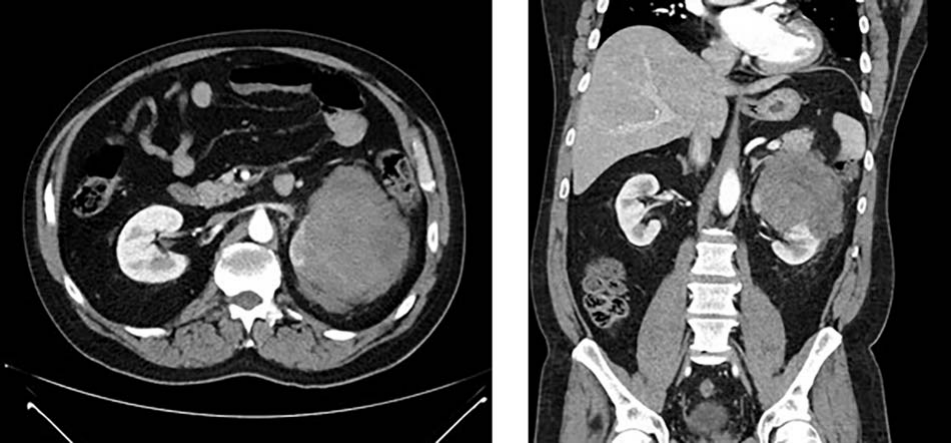
Left renal mass measuring 11 × 9 × 7cm in transverse (left) and coronal (right) planes.

**Figure 2:**
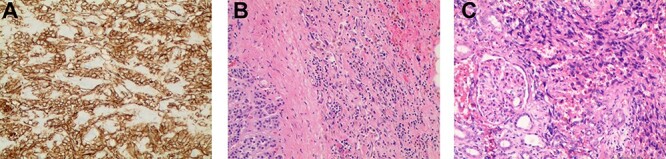
(**A**) Immunostaining for CD31 (100×) strongly marked lesional cells, supporting the diagnosis of AS and excluding sarcomatoid RCC; (**B**) routine H&E-stained section (100×) demonstrating an infiltrative pattern sarcomatoid tumor with variable cellularity and characteristic slit-like spaces, including red blood cells, characteristic of AS; benign adrenal tissue is visible on the left; (**C**) routine H&E-stained section (100×x) demonstrating angiosarcomatoid pattern with benign glomeruli.

## CASE DESCRIPTION

A 52-year-old, previously healthy, Caucasian male presented to the emergency department (ED) with chief complaints of gross hematuria, abdominal pain, vomiting, diarrhea and left flank pain for 3 days. He had neither family history of cancer nor history of exposure to ionizing radiation, arsenic, thorium dioxide or vinyl chloride. He reported exposure to chemical tankers 10 years prior and had recent occupational exposure to paint-thinning agents. Urinalysis on admission showed large blood with later cytology significant for atypical epithelioid cells concerning for neoplasm of the kidney or bladder. Subsequent contrast-enhancedcomputed tomography (CT) of the abdomen revealed an 11-cm left renal mass, 2-cm para-aortic lymphadenopathy and possible invasion of the tail of the pancreas (Fig. 1). He was then referred to urology for evaluation and to discuss treatment options.

After discussing options for treatment, surgical management was deemed most appropriate. He subsequently underwent open left radical nephrectomy and adrenalectomy with partial ureterectomy and left para-aortic lymphadenectomy. Intraoperatively, the pancreas was found to be uninvolved. There were no intraoperative or immediate post-operative complications.

The resected tissue was stained with routine hematoxylin–eosin (H&E) and was immunostained with CD31 (Fig. 2a–c). Histological examination of the resected left kidney and adrenal gland revealed high-grade, multifocal AS with sarcomatoid and rhabdoid features and extensive tumor necrosis. The margins of the ureter, renal vasculature and soft tissue were negative for malignancy. The sarcoma involved the tissue surrounding the adrenal gland but abutted the adrenal capsule. The tumor was staged as Stage IV, Grade 3 and T3N1M0. Examination of the resected periaortic lymph node revealed AS with involvement of one lymph node and adjacent perinodal soft tissue.

**Figure 3:**
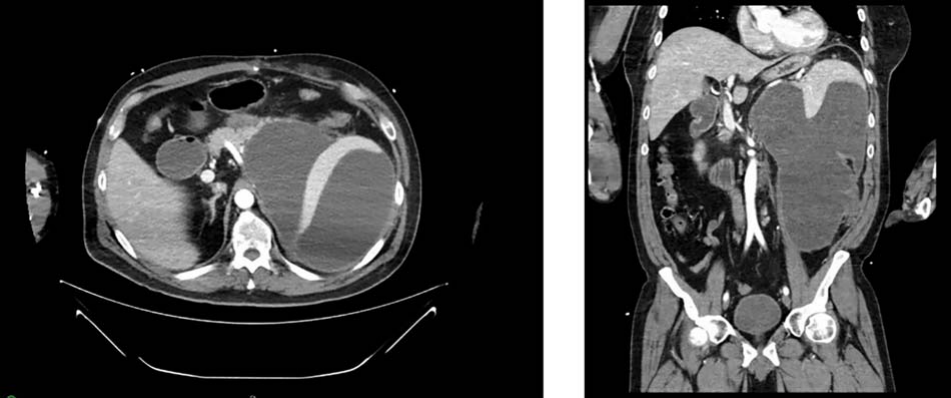
Appearance of effusion in the left retroperitoneal space in transverse (left) and coronal (right) planes on CT; it can be seen encroaching on the spleen; subsequent cytology ruled of malignant effusion.

Two weeks post-resection, the patient was recovering as expected. At 4 weeks post-resection, radiation oncology recommended 7 weeks of intensity-modulated radiation therapy to both the surgical site and at-risk nodes identified on CT. Six weeks post-resection, the patient presented to the ED with nausea and vomiting. Abdominal CT revealed a retroperitoneal effusion at the surgical site (Fig. 3). At this admission, the patient also tested positive for COVID-19, delaying drain placement and his radiation treatments for 2 weeks.

**Figure 4:**
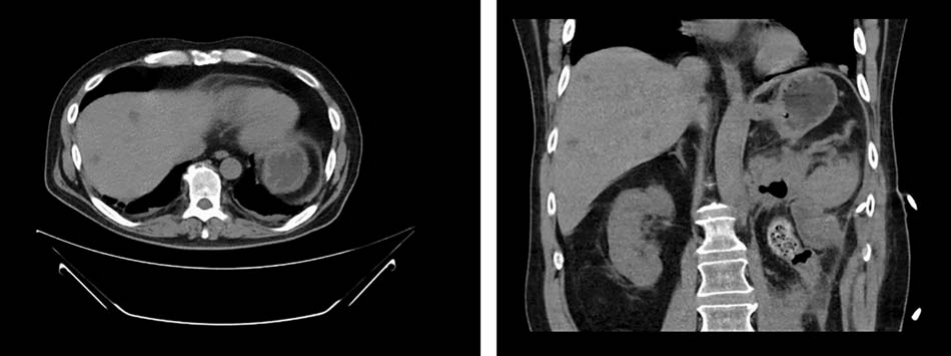
First appearance of multiple liver lesions suspicious for metastasis in transverse (left) and coronal (right) planes on CT; reduction of retroperitoneal effusion can also be seen.

Serial CT scans at 10 and 12 weeks post-resection revealed reduction of the retroperitoneal abscess and appearance of liver lesions concerning for metastasis (Fig. 4). The patient eventually completed his radiation course with no additional problems.

**Figure 5:**
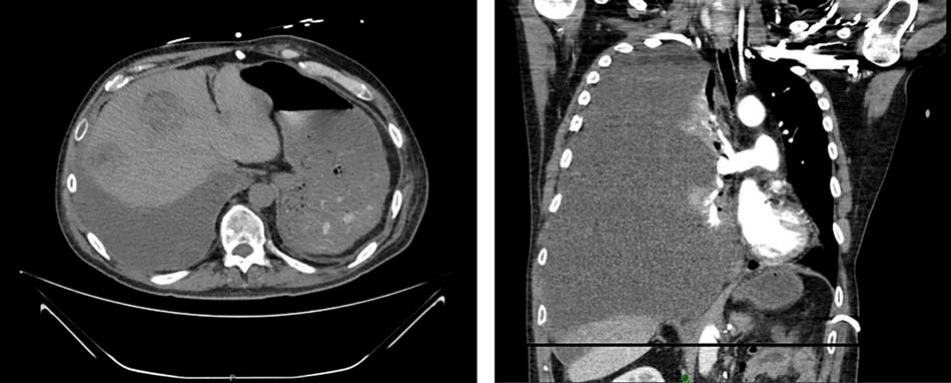
CT demonstrates growth of effusion into the entirety of the right hemithorax with mass effect shifting all thoracic contents to the left (left); later cytology ruled out malignant effusion; substantial increase in liver metastatic lesions can also be seen when compared to previous images from 2 months prior.

Fourteen weeks post-resection, the patient presented to the ED for symptoms of pneumonia and again tested positive for COVID-19. Chest X-ray showed evidence of small, right-sided pleural effusion. He was discharged for home quarantine. Ten days later, he returned to the ED with worsening shortness of breath and chest pain. CT showed a nonmalignant effusion filling the entire right hemithorax as well as significant enlargement of liver lesions (Fig. 5). He was discharged a week later following symptomatic improvement but experienced an exacerbation of respiratory symptoms soon after and was readmitted.

Over the next 2 weeks, his respiratory distress worsened, requiring increased oxygen supplementation. Oncology recommended starting Taxol for concern of liver metastasis, but this was not started due to the patient’s rapid deterioration. He subsequently required intubation due to cardiopulmonary arrest and was discharged to inpatient hospice. He expired the following day, surviving a total of 200 days from the day of tumor resection.

## DISCUSSION

The overall survival for AS is reported to range from 6–16 months after diagnosis, with a median age of diagnosis of 60–71 years [1]. This patient was significantly younger than this range at diagnosis and survived just over 6 months after presentation. Although he did not have any of the classic exposures associated with AS, he did have other concerning occupational exposures [6].

Most of the knowledge surrounding primary renal AS originates from case reports, which suggest the most important factors in predicting survival are tumor size and metastasis at presentation. Tumors <5 cm may have better odds of long-term survival following resection, but further studies are necessary to create a more reliable cut-off point [7]. This hypothesis is supported by this case, given the patient’s large tumor size (11 cm) and short survival after resection.

Routine screening for renal cancers is not recommended for most, apart from patients who have heritable disorders which increase their risk [8]. Thus, renal masses are often found incidentally during unrelated work-up. This inadvertent detection often identifies renal cancers before they are symptomatic, increasing the odds of long-term survival.

Lastly, this case highlights the barriers to care which patients faced during the COVID-19 pandemic. Many patients with multiple co-morbidities were unable to receive adequate or continuous care due to concerns surrounding COVID-19. Positive tests often prohibited patients from receiving hospital admission or outpatient treatment in favor of home quarantine. For this case, positive COVID-19 testing delayed care on two occasions; it caused a delay of radiation treatments and in the treatment of pleural effusion which could have prevented its subsequent progression. Although this patient’s overall survival was likely minimally affected, given his tumor size and presence of metastasis, this case provides examples of how care for many patients across the world may have been compromised because of the COVID-19 pandemic.
